# Bacteriophages and their unique components provide limitless resources for exploitation

**DOI:** 10.3389/fmicb.2024.1342544

**Published:** 2024-02-06

**Authors:** Christine M. Szymanski

**Affiliations:** Department of Microbiology, Complex Carbohydrate Research Center, University of Georgia, Athens, GA, United States

**Keywords:** bacteriophages, glycan binding proteins, glycoside hydrolases, glycosyltransferases, modified nucleobases, self-assembling nanoparticles, vaccines

## 1 Introduction

Since the original discovery of bacteriophages by William Twort in 1915, and Félix d'Hérelle in 1917, there have been numerous reports describing the use of these “filterable viruses” in phage therapy, and interest in this area continues to grow as the frequency of human infections with multidrug resistant bacteria escalates. In addition, a growing list of significant historic moments in science were only made possible through the study of phages. These achievements include the first demonstration that DNA is the genetic material [leading to the Nobel Prize in Physiology or Medicine in 1969 (Hershey and Chase, 1952)]; the first SDS-PAGE separating phage T4 proteins performed by Laemmli [and among the top 10 most cited papers of all time (Laemmli, 1970)]; the first DNA sequencing ever to be performed by Sanger on the phage φX174 genome [and leading to his second Nobel Prize in Chemistry in 1980 (Sanger et al., 1977)]; the first isolation of a bacterial restriction enzyme capable of cleaving phage T7 DNA [and a joint Nobel Prize in Physiology or Medicine in 1978 (Smith and Wilcox, 1970)]; and the first description of gene editing through the use of a phage defense mechanism known as CRISPR/Cas9 [and again a Nobel Prize in Chemistry in 2023 (Barrangou et al., 2007; Jinek et al., 2012)]. These and several additional studies have emerged demonstrating that phages are an invaluable resource to scientists and that their individual components have a plethora of functions beyond the usage of intact phages for bacterial therapy and gene transduction [for an excellent review, see Salmond and Fineran (2015)]; although new applications for phages even in gene cloning and expression have emerged through the discovery of phage N15's ability to deliver linearized vectors into bacteria, and now also engineered for use in eukaryotic cells (Wong et al., 2023). Additional phage applications include the use of their receptor binding proteins for bacterial detection, or engineering intact phages for bacterial viability reporting [using phage cocktails or synthetic approaches to expand host ranges (Sun et al., 2023)], and even uses in tumor detection, targeted therapeutic delivery (Shen et al., 2023), or reducing cancer progression (Sanmukh et al., 2021a,b); the exploitation of filamentous phages for phage display (Franca et al., 2023) traditionally for antibody screening and more recently for antibacterial development (Zhao et al., 2023); exogenous addition of phage endolysins (Fischetti, 2011; Cahill and Young, 2019; Abdelrahman et al., 2021) as a method for killing of Gram-positive (Mursalin et al., 2023), and more recently, Gram-negative microbes (Gondil et al., 2020) [together with outer membrane permeabilizers (Kocot et al., 2023)]; phage-based vaccines (more on this below) (Palma, 2023); and the use of phage depolymerases as adjuvant therapy to disperse microbial biofilms (Topka-Bielecka et al., 2021). Bacteria have also retained phage proteins such as their toxins (typically in lysogenic conversion) to help with dissemination (Kumar et al., 2020), or permanently adapted phage tail-like structures in Type VI secretions systems for injection of toxic effectors into eukaryotic or bacterial hosts (Leiman et al., 2009), or co-opted holins coupled with cell-wall editing enzymes in Type X secretion systems (Palmer et al., 2021).

In this opinion article, I will discuss re-emerging trends to exploit phages for glyco-tool discovery, glycan display and vaccine development. Although phage display was first described in 1985 by George Winter (who subsequently received the Nobel Prize together with Gregory Smith in 2018), the use of phages to display sugars, either linked enzymatically or chemically, is more recent. Also, since phages are genetically tractable and three-dimensional, they provide many advantages that inert glycan arrays cannot provide. Similarly, one of the first reports of antibody Fab binding to a carbohydrate, the Salmonella O-antigen oligosaccharide, was published in the seminal paper by Cygler et al. (1991) and phage display was subsequently used to select for the infamous carbohydrate-binding monoclonal antibody Se155-4 (Deng et al., 1994). It was 6 years later that the same O-antigen was crystallized with the Salmonella phage P22 tailspike protein (TSP), a protein that also possessed endoglycosidase activity (Steinbacher et al., 1997). However, this was not the first report of a phage polysaccharide depolymerase, these activities were already described in the 1950s (Maxted, 1952; Adams and Park, 1956). Both phage binding to carbohydrates and hydrolase/lyase activities have been described on an individual lectin/enzyme basis, similar to the P22 TSP example given above. However, functional assays for screening for broad phage activities within a population or data mining through genome databases is only now being considered.

## 2 A bottomless toolbox for glycobiologists

**Figure 1:**
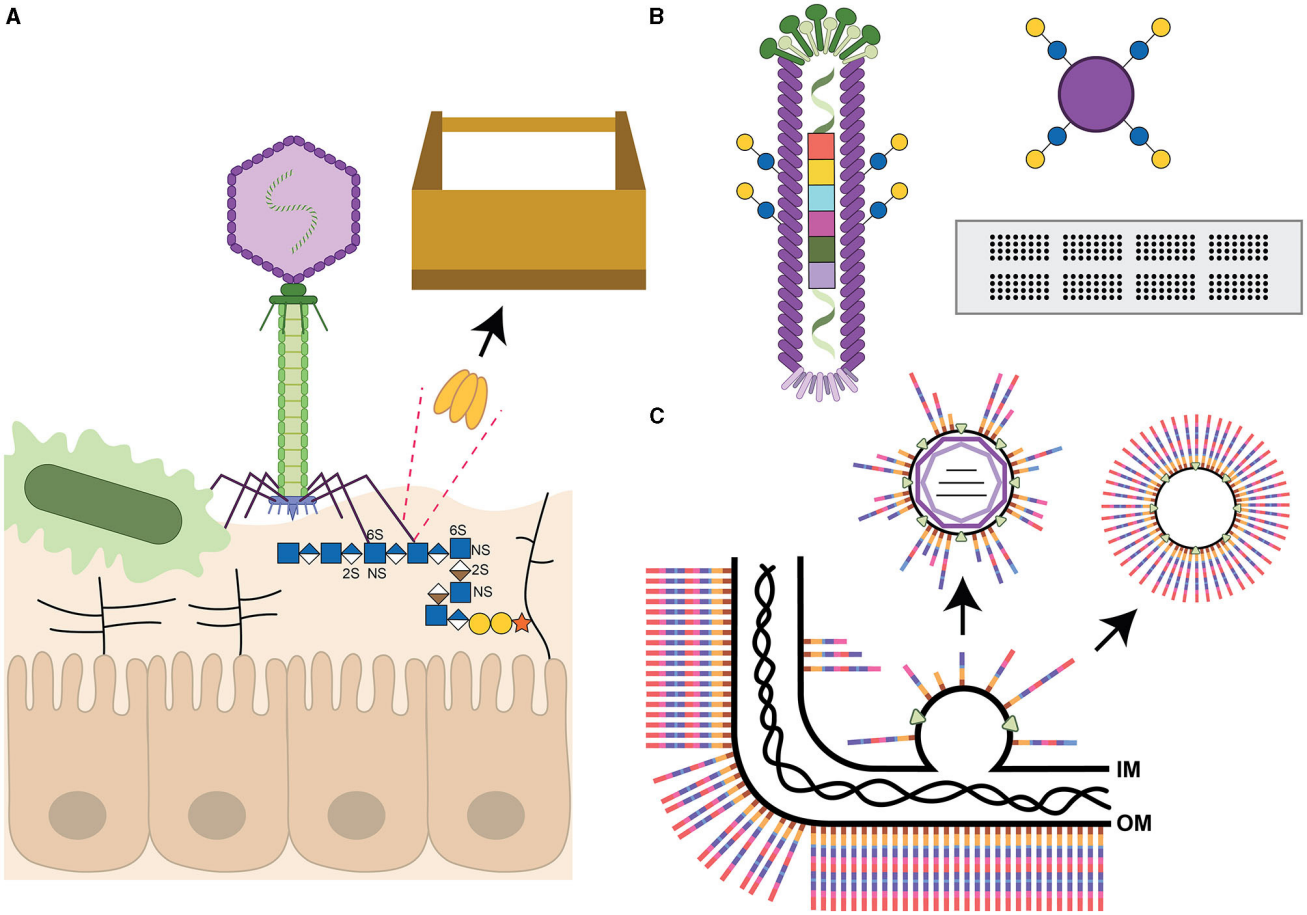
Summary of bacteriophage components described in this review. **(A)** T4-like phage (not drawn to scale) with carbohydrate-binding (lectin) tail fibers adhering to the capsular polysaccharides of a microbe, and likely possessing glycoside hydrolase/lyase activity to cleave the receptor to gain access to the bacterial cell wall. The tail fiber is also shown to interact with heparan sulfate glycosaminoglycans (GAG, represented as horizontal lines except for the GAG interacting with the phage) extending from proteins on host epithelial cells (according to Green et al., 2021). The dotted lines highlight one of the receptor binding proteins, depicted as a trimer, as a candidate for the toolbox of reagents useful for glycan detection or cleavage. **(B)** Filamentous phage covalently modified with glucose and galactose disaccharides as part of the liquid glycan array (LiGA) technology. The phage genome has also been tagged with a barcode to enable identification of adherent phages from deep-sequencing data (according to Sojitra et al., 2021). This glycan display platform is compared to two commonly used methods, Luminex beads modified with the same sugars as shown for the phage, and glycan arrays on glass slides. **(C)** Assembly of a double-stranded RNA Cystovirus demonstrating that the viral envelope is derived from the host inner membrane (IM) by an unknown self-assembly process and may contain intermediates from bacterial glycoconjugate biosynthesis pathways. The bacterial outer membrane (OM) with a generic full-length polysaccharide is shown with the peptidoglycan layer underneath. The rightmost arrow depicts the possibility of engineering self-assembling lipid vesicles with defined carbohydrate structures. All carbohydrates are drawn using the Symbol Nomenclature for Glycans (Neelamegham et al., 2019). Iduronic acid (divided brown diamond), galactose (yellow circle), glucose (blue circle), glucuronic acid (divided blue diamond), *N*-acetylglucosamine (blue square), and xylose (orange star). S indicates sites of sulfation with arbitrary linkage site.

I recently attempted to summarize the complexity in the composition of bacterial surface glycans in Gram-negative, Gram-positive and acid-fast microbes, like *Mycobacterium tuberculosis* (Szymanski, 2022). I subsequently used *Campylobacter jejuni* as the model to emphasize that each bacterial strain within this Gram-negative species possesses separate pathways for the biosynthesis of several glycoconjugate structures including *N*- and *O*-linked glycoproteins, capsular polysaccharides (CPS) and lipooligosaccharides (Szymanski, 2022). In addition, the set of enzymes required for synthesis of the three latter conjugates differs from strain to strain resulting in different carbohydrate structures and serotypes; and even within a single strain, many of the enzymes can vary in expression (on or off) leading to the possibility of one in >1,000 different glycan compositions (for CPS alone) on each individual cell. Now considering that (1) NMR has been used to solve glycoconjugate structures for a miniscule number of bacterial strains to inform our understanding of the vastness of unique monosaccharides that the domain of Bacteria are capable of synthesizing, and (2) phages predominantly express receptor binding proteins recognizing specific bacterial surface glycan structures and glycoside hydrolases/lyases capable of degrading polysaccharides in order to gain access to the bacterial cell surface (Simpson et al., 2015), it is obvious to conclude that the estimated 10^31^ phages on Earth (Mushegian, 2020) provide an enormous repertoire for discovering proteins (glyco-tools) involved in glycan binding and degradation as a consequence of this evolutionary arms race (Figure 1A). However, the possibilities do not end with simply the identification of novel lectins and enzymatic functions [now also including glycosyltransferases modifying capsids and tail tubes (Freeman et al., 2023)], since many enzymes can also be manipulated to maintain the lectin binding domain while inactivating the enzymatic activity, or optimized through methods such as phage display to exhibit improved activity. This would provide the scientific community with a limitless number of reagents for bacterial glycan labeling and structural characterization to better define the carbohydrate receptors necessary for phage therapy and enable researchers to screen pathogenic isolates to assess the conservation of specific glycan structures for glycoconjugate vaccine design.

Furthermore, phage genomes are not static, but rather quick to acquire new mutations and recombination events and are capable of also adapting to bind to human glycans, such as fucosylated mucins, when coevolved together with the bacterial host and gut-on-a-chip environment (Chin et al., 2022). The binding of human glycans was also observed by Green et al. when they demonstrated that the phage tail fiber could bind to human heparan sulfate proteoglycans co-localizing the phage to the epithelial cell surface in proximity to the *Escherichia coli* bacterial host (Figure 1A). The authors concluded that their new findings provide the opportunity to target phages to mucosal surfaces to selectively remove pathogens from these sites (Green et al., 2021). It will also be interesting to explore whether the phage expresses two different phage tail fibers with different carbohydrate specificities, or whether these fibers possess two separate glycan binding sites, similar to the CTX-phage encoded cholera toxin B-subunit (Heim et al., 2019). These observations further expand the toolbox to include reagents of value for eukaryotic glycobiologists alike.

## 3 Phage glycan display

Phages can serve as templates for glycan display to allow for screening of scrambled protein sequons permissible for glycosyltransferase recognition (Durr et al., 2010), or to rapidly screen for genetically altered transferases capable of protein modification. More recently, phages have been used for density-controlled, multivalent display of varying glycan structures, as an alternative to glycan arrays, to screen glycan binding interactions (Sojitra et al., 2021; Lin et al., 2023). To accomplish this, genetically bar-coded M13 filamentous phages are chemically functionalized with different glycan structures in a process known as LiGA (for liquid glycan array, shown in Figure 1B in comparison to more commonly used glycan arrays). Incubation with a cocktail of phages, each displaying a different carbohydrate structure, allows investigators to characterize glycan binding preferences of purified proteins, or glycan recognition by adhesins on specific cell types, or glycan enrichment correlated with known organ functions in mouse models (Sojitra et al., 2021; Lin et al., 2023). Another application of this method would be to investigate bacterial binding to various glycan structures which very few laboratories have been able to assess through the use of standard glycan arrays (Day et al., 2016; Semchenko et al., 2019; Sanchez et al., 2023).

## 4 Vaccine development—Manipulation of nucleobases

Many researchers have shown that the successful breakthrough in the use of mRNA vaccines to treat SARS CoV-2 viral infections hinged on the use of the modified nucleobase, N1-methyl-pseudouridine (Morais et al., 2021; Nance and Meier, 2021). And Karikó and Weissman subsequently received the Nobel Prize in Physiology or Medicine in 2023 for demonstrating the benefits of this modified nucleoside base in protein expression and immune recognition (Karikó et al., 2008). The application of this new mRNA vaccine platform and the encapsulation into lipid nanoparticles (see below), has caused an explosion in vaccine development targeting a number of infectious agents, and phages may provide new alternatives for the biopharmaceutical industries. For example, there are currently 40 DNA and 150 RNA modifications known, and many of these are found in phages (Nielsen et al., 2023). Furthermore, we and others have identified several new single-stranded (ss)RNA and double-stranded (ds)RNA phages and their RNA compositions have yet to be explored (Crippen et al., 2021; Thongchol et al., 2023). Also, some phages, including the *C. jejuni Firehammervirus* phages, possess mechanisms to precisely exchange canonical nucleotides with non-canonical nucleotides with 100% efficiency into an existing DNA strand (Crippen et al., 2019). Such enzymes would have great utility in creating novel DNA/RNA templates for functional studies and potentially engineering mRNA vaccines against a variety of pathogens more efficiently and/or with novel properties.

## 5 Vaccine development—Self-assembling lipid nanoparticles

*Pseudomonas syringae* phage ϕ6 was the first enveloped dsRNA Cystovirus identified more than 50 years ago (Gottlieb and Alimova, 2023) and has served as the model phage to understand phage RNA biosynthesis, packaging and host lipid bilayer recruitment. Reminiscent of eukaryotic viruses, the phages use protein-triggered membrane fusion for entry into their bacterial hosts (Bamford et al., 1987) and the phages exit surrounded by host phospholipid bilayers from the inner membrane (Heymann, 2023). These envelopes are generated through the insertion of one specific phage protein (that must be expressed together with its chaperone) and vesicles of similar buoyant density as ϕ6 phospholipid precursors can be generated independently through the sole expression of these proteins (Johnson and Mindich, 1994). More recently, *P. aeruginosa* enveloped RNA viruses were discovered (Yang et al., 2016; Antonova et al., 2023), followed by similar viruses described by our group targeting *Acinetobacter radioresistens* (Crippen et al., 2021). Not only is the mechanism of membrane acquisition and assembly around the RNA-core require further elucidation for these Cystoviruses (Sun et al., 2017), but it is currently unknown whether the glycolipid intermediates normally assembled on the bacterial inner membrane are being excluded or whether the bacterial membranes can be manipulated to include specific glycans to target immune cell receptors or to include glycan antigens of interest (Crippen et al., 2021) (Figure 1C). It would be interesting to determine whether naturally enveloped phages such as those described for *Pseudomonas*, and more recently for *Acinetobacter*, can be engineered to form self-assembling lipid nanoparticles displaying specific bacterial polysaccharides (Figure 1C). This would be reminiscent of glycoconjugate expressing outer membrane vesicles (OMVs) that have been successfully used in many studies (Dull and McIntosh, 2012; Sinha et al., 2015; Chen et al., 2016; Liu et al., 2016; Price et al., 2016; Banerjee et al., 2023). There would be several added advantages to using the enveloped phages including the presence of a phospholipid bilayer rather than the use of an endotoxin containing outer membrane, homogeneous particle sizes, facile generation of particles from bacterial fermentor growth, etc. Similarly, these lipid nanoparticles can potentially be engineered to carry different cargo including select protein antigens or modified mRNA, perhaps by taking advantage of some of the mechanisms that phages have evolved to alter their own nucleoside bases. Not discussed, but equally interesting, there are extensive efforts in using well-characterized phage proteins such as the Salmonella phage P22 capsid as a carrier, reactor or vaccine (Wang and Douglas, 2022; Essus et al., 2023), or the phage Qβ system (Sungsuwan et al., 2022), among others (Yuan et al., 2023).

## 6 Conclusion

There is no doubt that bacteriophages will continue to inform our understanding of a multitude of biological and chemical processes, and lead to key discoveries and additional Nobel Prizes for years to come. Since Félix d'Hérelle's first realization that phages can be used to manage microbial infections, researchers have continued to make improvements in this approach and are also exploring the use of several phage components as their unique functions are being realized. In this article, I emphasized that phages will act as a constant source of reagents for glycobiologists since it is known that as bacteria adapt and change their carbohydrate structures for phage evasion, phages alter the specificities of their receptor binding proteins and polysaccharide depolymerases for bacterial infection. Similarly, phages will also be more commonly used in glycan display platforms to better understand the binding specificities in carbohydrate-protein interactions. Lastly, enveloped phages have the potential to replace OMVs, lipid nanoparticles and virus-like particles in various vaccine platforms and I anticipate this will be a topic for future development.

## Author contributions

CS: Conceptualization, Writing—original draft, Writing—review & editing.
